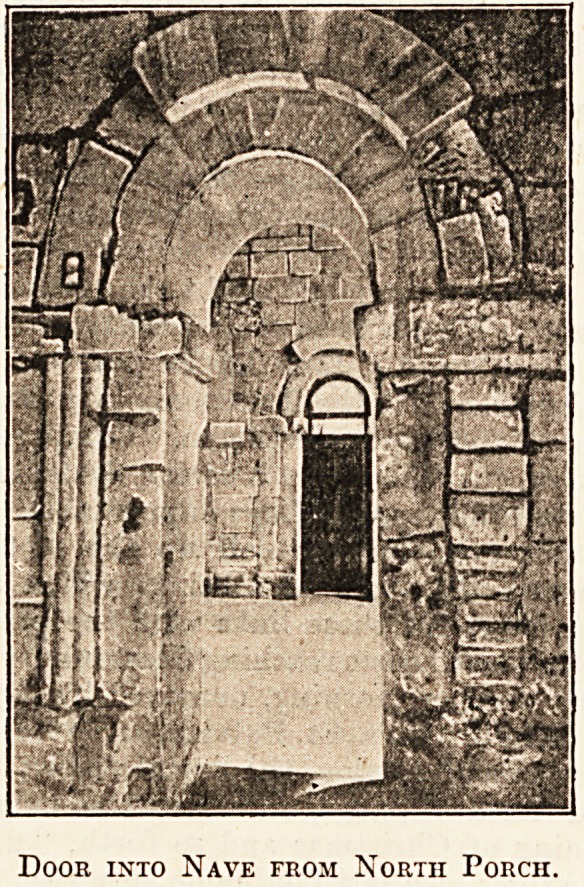# Architecture and History

**Published:** 1908-01-18

**Authors:** 


### The Practitioner's Relaxations and Hobbies.

### ARCHITECTURE AND HISTORY.

A fellow-student of the writer, after a month's hard midwifery work amongst the poor in London, betook himself to picture galleries for a whole week, as a corrective to the squalor and sordid surroundings with which he had been in contact. So in later

Whitby Abbey from North-West.

years the tired general practitioner may feel the need of some tonic, intellectual and spiritual, to lift him up: some alterative to remove him from the dull routine of coughs and quinsies, of bad debts and broken nights, to some more ideal level.

When settled at Whitby and waiting for practice the writer found much rest and relaxation in gardening, a pursuit new and interesting to a Londoner. As practice grew and with it cares and worries, some new outlet became necessary, and a bent was given in the direction of architecture and then English history by a little incident. A Leeds-bred lady who visited Whitby would in the early morning seek the solitude of the ruined abbey for her private devotion. Bold and striking in its position, no better example of the Early English style can be wished for and as you note the sweet symmetry of the lancet windows, the dog-tooth mouldings of the choir, and the clean-cut rose-window of the transept, you are learning for the remainder of your days a lesson in taste and purity of conception; and this impression will be ready to hand (as it were) when you look up at the majestic proportions and silvery tints of the Five Sisters' Window at York, or the delicate fretwork of the Confessor's Chapel at Westminster. There is a charm about architectural study in that you pick up your information bit by bit; you associate it with holiday occasions and places, perhaps with delightful ruins in a setting of rustic peacefulness; you never get to the end of it, or feel you have quite mastered it, and that there is nothing more left for you to learn about it. And what you have once learnt is always useful in examining other buildings afterwards. Many a village church has thus a sermon in stone built into it. There is a mild species of excitement as you step in silently through some venerable door, hardly knowing what relics of bygone ages may be found within. You may learn a spiritual lesson from some holy-water stoup, some chair for penitents, a squint-window or an altar-stone: you may find yourself kneeling in silent homage beside the brasses at Stoke d'Abernon or the Crusaders' tombs in the Temple.

And what a sermon might be preached on epitaphs! Eloquent in their brevity, as "O rare Ben Jonson" and "Jane Lister dear childe" at Westminster, or like the Runic crosses in Manxland recalling Danes and Northmen. Compared with these, the quiet monomania of the fly-fisherman is almost stale, flat, and perhaps unprofitable.

But not everyone can call up the sentimental reverie and the inspired musings which are the peculiar charm of these links with the past. For example: two tourists reaching Glastonbury's noble ruin one summer morning, unmoved by legends of Joseph of Arimathæa, St. Patrick, and St. Dunstan, the very Isle of Avalon, the burial-place of the renowned Arthur and Guinevere, the holy thorn blossoming at Christmas and so forth, "did" the place to their own satisfaction on this wise. Stand-

Netley Abbey, Southampton.

ing just within the entrance-gate one remarked, "Well, we have seen Glastonbury." "Yes," replied his friend; and they were gone within two minutes of entering the sacred precincts. But with true poetic fervour in one, who would not give all else to spend a summer evening there at Stoke Poges, where Gray composed his matchless "Elegy," or to haunt that earliest Norman chapel in the Tower where the Conqueror attended service and Mary was betrothed to Philip of Spain; or, saddest spot, St. Peter-ad-Vincula Church, where were laid the bleeding relics of noblemen and queens, Chief Justice Jeffreys beside Monmouth, and Thomas Cromwell beside Fisher and More? Who would neglect a chance of making the pilgrimage to Stratford to read the blessing and curse upon the tomb of him who con-

Door into Nave from North Porch.

ceived Hamlet and Lady Macbeth, whom parish registers show to have been tempted like as we are?

There is so little remaining to our own time of Roman or Saxon work that it is simplest to begin with some Norman example, as Christchurch Priory (Hants), Adel near Leeds, St. Bartholomew's Smithfield, Peterborough, Ely, Norwich, or stately Durham, and then trace on the transition to Early English in Southwell, Whitby, Fountains Abbey, or the Temple Church. Very fine examples of the latter are found in the cathedrals at Worcester, Wells, Salisbury, and Lincoln, but it is easier to grasp the main features in some wayside church such as Bishop's Cannings near Devizes, or lonely Morwenstow on the North Cornish coast, with its mossy lich-gate and memories of its poet-vicar of bygone days. A very good start can be made by a short stay in Normandy, and such towns as Caen, Falaise, Flers, Domfront, and (if time permits) Bayeux and Mont St. Michel will fill anyone with admiration for the renowned builders who planted our island with churches which are a joy for ever.

It is easy then to trace back the rude efforts of the Saxons by a close study of the little church of St. Lawrence at Bradford-on-Avon, or Barnack, Brixworth, and Earl's Barton in Northamptonshire. For what the Romans did we must look to such towns as Leicester, York, and Bath, St. Martin's at Canterbury, the more extensive remains at Silchester, and Hadrian's wall in the north. When the simpler styles are familiar, it is an easy passage on to the later ones, as in the churches of Hedon and Patrington, the King and Queen of Holderness; the chapter-house at Southwell, the cloisters at Norwich, and the nave at York and Exeter, which show the Decorated style at its best; and we shall find examples of the Perpendicular period in the parish churches at Rotherham and Newark, the cathedrals of Gloucester and Canterbury, Henry VII.'s chapel at Westminster, Windsor, and many other places. Two or three or more styles may be seen in the same building, as at Beverley Minster, Barnack, Southwell, Carlisle, Ripon, and St. Albans.

The history bound up with these buildings and periods is by no means their least interesting feature. For instance, Westminster Abbey is a prose poem, a library, and a museum in itself. And the study of them, if reinforced by some illustrated work such as Green's *Short History*, may lead on to the culture of English History or Antiquities in general.

## Figures and Tables

**Figure f1:**
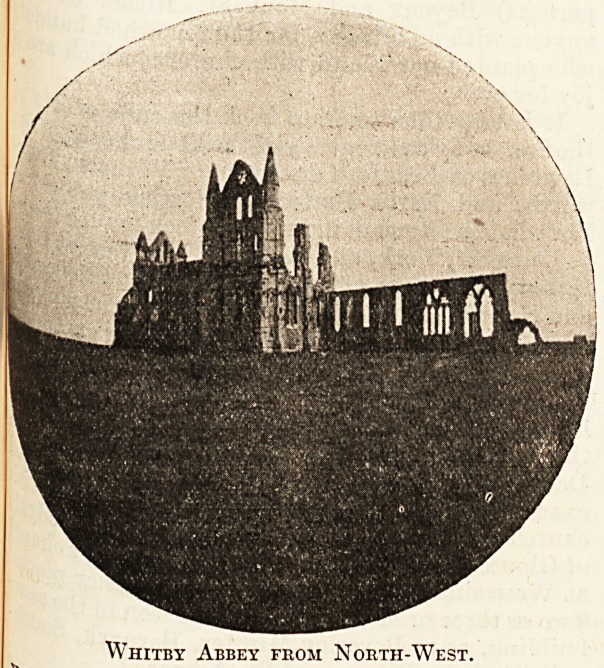


**Figure f2:**
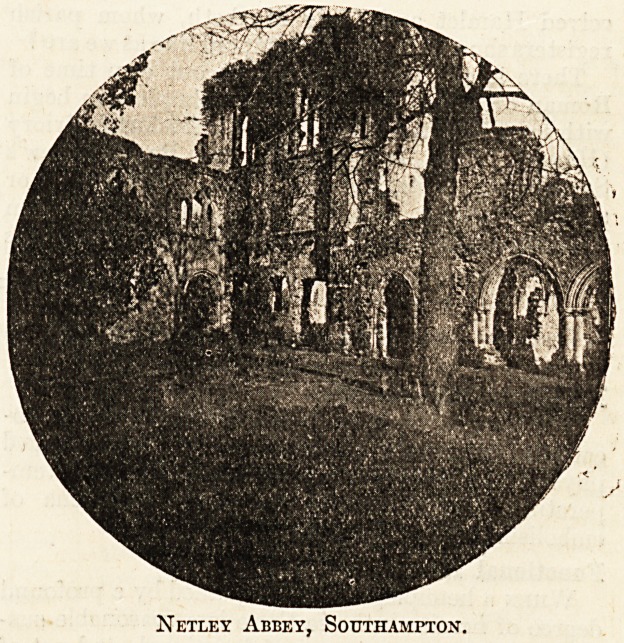


**Figure f3:**